# A case of azoospermia in a non-destructive testing worker exposed to radiation

**DOI:** 10.1186/s40557-017-0190-z

**Published:** 2017-08-10

**Authors:** Jaechan Park, Sanggil Lee, Chulyong Park, Huisu Eom

**Affiliations:** Occupational Safety and Health Research Institute, KOSHA, Ulsan, Korea

**Keywords:** Azoospermia, Cytogenetic dosimetry, Non-destructive testing, Male infertility, Occupational disease, Radiation

## Abstract

**Background:**

Interest in radiation-related health problems has been growing with the increase in the number of workers in radiation-related jobs. Although an occupational level of radiation exposure would not likely cause azoospermia, several studies have reported the relation between radiation exposure and azoospermia after accidental or therapeutic radiation exposure. We describe a case of azoospermia in a non-destructive testing (NDT) worker exposed to radiation and discuss the problems of the related monitoring system.

**Case presentation:**

A 39-year-old man who was childless after 8 years of marriage was diagnosed with azoospermia through medical evaluations, including testicular biopsy. He did not have any abnormal findings on biochemical evaluations, other risk factors, or evidence of congenital azoospermia. He had been working in an NDT facility from 2005 to 2013, attaching and arranging gamma-ray films on the structures and inner spaces of ships. The patient’s thermoluminescent dosimeter (TLD) badge recorded an exposure level of 0.01781 Gy for 80 months, whereas results of his florescence in situ hybridization (FISH) translocation assay showed an exposure level of up to 1.926 Gy of cumulative radiation, which was sufficient to cause azoospermia. Thus, we concluded that his azoospermia was caused by occupational radiation exposure.

**Conclusion:**

The difference between the exposure dose records measured through TLD badge and the actual exposure dose implies that the monitor used by the NDT worker did not work properly, and such a difference could threaten the health and safety of workers. Thus, to protect the safety and health of NDT workers, education of workers and strengthening of law enforcement are required to ensure that regulations are strictly followed, and if necessary, random sampling of NDT workers using a cytogenetic dosimeter, such as FISH, should be considered.

## Background

According to a 2012 report of the Nuclear Safety and Security Commission, 5606 workplaces handle radiation-generating devices and radioactive isotopes in Korea, and the number of workers in radiation-related jobs has been estimated to be 42,226, which is increasing every year, with interest in radiation-related health problems simultaneously increasing [1].The radiation at these workplaces influences the health and safety of workers, causing various conditions, such as cancer and genetic and hereditary effects [2, 3]. Radiation exposure can also possibly cause male infertility, which is the failure to achieve pregnancy after 12 months of intercourse [4] and is known to result from combined abnormalities in sperm count, motility, and morphology [5]. Azoospermia is the absence of spermatozoa on high-powered microscopic examination of at least two samples of seminal fluid [6]. The prevalence of azoospermia has been estimated to be approximately 1% among all men and 10%–15% among infertile men [7].

Exposure to ionizing radiation occurs in diagnostic and therapeutic medicine as well as in the industrial setting [3], and it is also known to cause damage to cells and non-lethal transformation of cells that can result in functional impairment of the testes, as these organs are very radiosensitive [8]. Although an occupational level of radiation exposure would not likely cause azoospermia [9], several previous studies have reported that therapeutic and accidental radiation exposure above certain values could induce azoospermia. We herein present a case of azoospermia in a patient who was exposed to radiation in a non-destructive testing (NDT) facility and discuss the problems of the related monitoring system.

## Case presentation

### Patient

Thirty-nine-year-old man.

### Chief complaint

Infertility for 8 years after marriage.

### Present illness

The patient did not use birth control methods and did not have any sexual problems, such as erectile dysfunction, throughout his married life. However, he did not have a child during 8 years of marriage. Thus, he visitedan infertility clinic in July 2013. His wife did not have any fertility problems; however, he was diagnosed with azoospermia associated with Sertoli cell–only syndrome (SCOS).

### Medical history

He had a medical history of hypertension.

### Social history

Ex-smoker (16 pack-years) and social drinker (one bottle of so-ju twice a week).

### Assessment of infertility

Our patient’s height was 173 cm and weight was 71 kg. Semen analysis indicated azoospermia in two semen samples. His follicle-stimulating hormone level was 19.84mIU/mL (reference range, 1.27–19.26 mIU/mL), luteinizing hormone level was 5.55mIU/mL (reference range, 1.24–8.62mIU/mL), and testosterone level was 2.51 ng/mL (reference range, 1.75–7.81 ng/mL). Genetic studies showed a normal 46 XY karyotype without Y chromosome microdeletions. Thus, testicular biopsy was performed to determine the cause of azoospemia, and he was diagnosed with SCOS.

### Occupational history

He worked in an NDT facility between August 2005 and June 2011. He usually worked day/night shifts for 3 weeks per month. However, he worked for 4 weeks a month, as the workload increased later. His primary job was radiographic testing, which used iridium-192 as the radiation source. He performed radiographic testing of ship structures in two heavy industries (2005–2008 and 2008–2010). His job involved attaching and arranging gamma-ray films on the structures and inner spaces of ships. According to work instructions, when an NDT equipment was being used, workers should be far away from the equipment that emits radiation and should be shielded from the radiation; however, the working environment, which had a narrow working space and restricted movement, and high workload did not allow enough protection from the radiation and time to move away from it. After July 2011, he changed jobs within the same field and with decreased use of radiation-emitting equipment.

### Assessment of radiation dose

Our patient had a thermoluminescent dosimeter (TLD) badge, which monitored and recorded his radiation exposure during work. According to the records, the cumulative radiation dose was 0.01781 Gy for 80 months (Table 1).

**Table 1:** Radiation exposure doses (mSv) measured through the TLD badge from 2005 to 2013^a^

Year	Jan	Feb	Mar	Apr	May	Jun	Jul	Aug	Sep	Oct	Nov	Dec
2005	-	-	-	-	-	-	-	0.10	0.10	0.10	0.10	0.10
2006	0.65	0.10	0.19	0.10	0.19	0.10	0.10	0.10	3.39	0.10	0.10	0.10
2007	0.10	0.10	0.10	0.10	0.10	0.10	0.10	0.10	0.10	0.10	0.10	0.10
2008	0.10	0.10	0.10	ND^b^	ND	ND	ND	ND	ND	ND	ND	ND
2009	ND	ND	ND	ND	ND	ND	ND	ND	ND	ND	ND	1.24
2010	ND	ND	ND	ND	0.19	NT	0.47	2.60	-	-	-	-
2011	-	-	-	-	-	-	-	-	0.11	0.11	1.93	0.12
2012	0.29	ND	0.56	0.72	0.79	0.14	ND	ND	0.11	ND	0.24	ND
2013	0.16	ND	0.52	-	-	-	-	-	-	-	-	-

^a^All contents were provided by Korea Foundation of Nuclear Safety

^b^ND, not detectable (lower than the minimum detectable level, <0.1 mSv)

In 2010, some workers at that NDT facility were diagnosed with leukemia and myelodysplastic syndrome. Because of this, all workers in that facility were required to undergo evaluation of radiation exposure dose through cytogenetic dosimetry. The dosimeters measured the exposure dose using specific genetic markers. Dicentric chromosome assay (DCA) and fluorescence in situ hybridization (FISH) translocation assay were performed in the workers [10–12]. Our patient’s radiation exposure dose was 0.882 Gy (95% confidence interval [CI], 0.597–1.183 Gy) in DCA and 1.913 Gy (95% CI, 1.358–2.591 Gy) in FISH at the time. In 2015, he underwent another exposure evaluation after the diagnosis of azoospermia. The radiation exposure dose was 0.491 Gy (95% CI, 0.219–0.822) in DCA and 1.926 Gy (95% CI, 1.349–2.624 Gy) in FISH (Table 2). Considering his exposure period and the fact that FISH accurately reflects long-term cumulative exposure, we concluded that the patient’s cumulative radiation exposure dose was 1.926 Gy.

**Table 2:** Assessment of radiation exposure dose (Gy) through cytogenetic dosimetry^a^

Date	Cytogenetic dosimetry (dose)	
	Dicentric chromosome assay (95% CI)	FISH translocation assay (95% CI)
Aug 2010	0.882 (0.597–1.183)	1.913 (1.358–2.591)
Dec 2010	0.876 (0.589–1.197)	1.848 (1.452–2.292)
Feb 2011	0.765 (0.479–1.076)	1.815 (1.200–2.509)
May 2011	0.750 (0.482–1.062)	1.813 (1.196–2.570)
Jan 2015	0.491 (0.219–0.822)	1.926 (1.349–2.624)

^a^All contents were provided by Korea Institute of Radiological Medical Sciences

## Discussion

This is a case of an azoospermic patient diagnosed with SCOS which was considered to be caused by occupational radiation exposure. There are many causes of SCOS, including ionizing radiation exposure [13–15]. Medical evaluation of our patient showed no reason to suspect other possible causes of SCOS; thus, considering his job, it is reasonable to assume that patient’s SCOS was caused by occupational radiation exposure.

We assessed the radiation exposure dose and evaluated whether he had fractionated or single exposure to determine whether his condition was caused by radiation, because it is known that spermatogenesis has higher radiation tolerance for single exposure than for fractionated exposure [16]. We inspected the patient’s job history and exposure measurement records (Table 1) and concluded that he was exposed to radiation protractedly for 80 months.

According to the report of the International Atomic Energy Agency, temporary male sterility could result from single short exposure and prolonged exposure doses of 0.15 and 0.4 Gy in a year, respectively, and that permanent male sterility could result from single short exposure and prolonged exposure doses of 3.5–6 and 2 Gy in a year, respectively [17].

Lu et al. showed that fractionated irradiation of 0.7–0.9 Gy was capable of causing oligospermia or azoospermia, but they assumed that spermatogenesis would recover in 1–1.5 years [18]. Howell and Shalet suggested that a fractionated dose of over 1.2 Gy could cause permanent infertility and a dose of over 2 Gy was very likely to cause permanent infertility [19].

Some studies suggested similar levels of exposure dose that cause azoospermia, and a number of studies about azoospermia caused by prolonged exposure to radiation were mainly conducted in patients undergoing radiotherapy for cancer treatment [20–22], and in these studies, some patient developed permanent azoospermia, whereas others experienced transient azoospermia at similar levels of exposure.

The findings of the above-mentioned studies suggest that radiation could induce permanent azoospermia at high exposure doses and that the threshold values vary from person to person, depending on each person’s distinct characteristics. Thus, a person will not necessarily experience azoospermia if exposed to a specific dose. In the present case, the patient was exposed to radiation of 1.926 Gy, which was among the threshold doses mentioned in previous studies, but this value is not usual in normal occupational exposure; the occupational exposure limit in Korea is 100 mSv for 5 years and 50 mSv in any given year. Furthermore, he did not have the evidence of congenital azoospermia or other risk factors. Therefore, it might be reasonable to conclude that his azoospermia was caused by occupational radiation exposure.

On estimating of patient’s exposure dose, we found that the exposure dose records measured through TLD badge did not reflect the actual exposure level. The Korean government has introduced laws about radiation and its control and monitoring of workers who might be exposed and of factories/facilities that handle radioactive material. However, workers might not wear the TLD badge or might use it inappropriately. Accordingly, assessment of radiation exposure with a TLD badge might underestimate the exposure dose, which means that the monitoring of radiation in NDT workers is not working properly. An institution report shows that the mean exposure dose of NDT workers has been increasing, despite the strict government regulations for NDT facilities [23]. It appears that the factories/facilities might be neglecting the regulations and underreporting exposure dose to circumvent government penalties.

Thus, to protect the safety and health of NDT workers, education of workers and strengthening of law enforcement are required to ensure that regulations are strictly followed, and if necessary, the use of cytogenetic dosimeter, such as FISH, for random sampling of NDT workers should be considered.

## Conclusion

In this case of azoospermia in an NDT worker who had been exposed to radiation, we noted a difference between the exposure dose on TLD measurement and the actual exposure dose of the patient. Although azoospermia is known to be rarely caused by normal occupational radiation exposure, our patient was exposed to a dose high enough to induce azoospermia in radiation workers. Thus, much attention and efforts are needed to correct this difference in exposure level and prevent health problems.

## Abbreviations

DCA: Dicentric chromosome assay
FISH: Florescence in situ hybridization
NDT: Non-destructive testing
SCOS: Sertoli cell–only syndrome
TLD: Thermoluminescent dosimeter
